# Welt im Umbruch – Deutschland braucht eine „Realpolitik im deutschen Interesse“

**DOI:** 10.1007/s12399-021-00858-5

**Published:** 2021-06-29

**Authors:** Alexander Gauland, Armin-Paulus Hampel

**Affiliations:** Fraktion der AfD, Deutscher Bundestag, Platz der Republik 1, 11011 Berlin, Deutschland

**Keywords:** Neue Realpolitik, Nationale Außenpolitik, Ehrlicher Makler, Wettstreit der Nationen, Interessenausgleich, New realpolitik, National foreign policy, Honest political agent, Competition of nations, Balance of interests

## Abstract

Deutschland steht auf der Weltbühne großen Herausforderungen gegenüber. Alte Mächte sind in der Krise und neue Machtzentren geben den Ton an. Regionale Konflikte wie in Afrika und Kriege wie in Syrien haben unmittelbare Auswirkungen auf Deutschland und Europa: wachsender Migrationsdruck und soziale Verwerfungen. Vor diesem Hintergrund schwankt die deutsche Außenpolitik zwischen Selbstverzwergung und dem Anspruch, „Weltmacht der Menschenrechte“ sein zu wollen. Als AfD fordern wir eine Rückbesinnung auf realpolitisches Handeln in der Außenpolitik.

## Abstract (en)

Germany faces major challenges on the world stage. Old powers are in crisis and new power centres are setting the tone. Regional conflicts as in Africa and wars as in Syria have a direct impact on Germany and Europe: increasing migratory pressure and social upheavals. Against this background, German foreign policy oscillates between self-deprecation and the aspiration to be a “world power of human rights”. As the AfD, we call for a return to *realpolitik* in foreign policy.

## Einleitung: Von guten und von bösen Mächten

Friedrich von Schiller schenkte uns in seinem 1804 vollendeten Drama *Wilhelm Tell* den folgenden Gedanken: „Es kann der Frömmste nicht im Frieden bleiben, wenn es dem bösen Nachbar[n] nicht gefällt.“ Kaum ein anderer Satz beschreibt das Weltgeschehen der Gegenwart zutreffender als dieser.

Böse Nachbarn, so glaubte man allerdings nach dem Mauerfall, gebe es in der nun anbrechenden Epoche, für die Francis Fukuyama bereits das Ende der Geschichte aufkommen sah, nicht mehr; sie seien vernünftigerweise in Gegenwart und Zukunft ebenso wenig vorstellbar wie die Existenz von Mauern, die Staaten trennen. Doch nicht nur die Geschichte geht weiter, sondern auch das ewige Austarieren von Interessen mit und manchmal gegen Staaten, die man in der heutigen Zeit nicht mehr so einfach in gute oder böse einteilen kann.

Insofern verfolgt die AfD-Fraktion im Deutschen Bundestag einen Kurs, den man durchaus als klassisch im Bismarck’schen Sinne, vor allem aber als pragmatisch bezeichnen muss. In einem unlängst verabschiedeten Strategiepapier bekennt sich die Alternative für Deutschland zu einer Realpolitik im deutschen Interesse und nimmt sich dafür die erfolgreichsten deutschen Außenpolitiker Otto von Bismarck, Gustav Stresemann und Hans-Dietrich Genscher zum Vorbild. Als größte Oppositionsfraktion im Deutschen Bundestag fordert die AfD eine Rückkehr zu einer eindeutig formulierten nationalen Außenpolitik. Die Vermischung außenpolitischer Ziele mit den Anliegen zahlreicher Interessensgruppen und NGOs, die demokratisch nicht legitimiert für sich mit Themen wie Klimapolitik, Gender und Frauenrechte weltweit ein politisches Mitspracherecht reklamieren, halten wir für falsch.

## Auch Europa partizipiert: Für eine Realpolitik im deutschen und europäischen Interesse

Die AfD bekennt sich zu einem Europa der Vaterländer im Sinne Charles de Gaulles. Dem Ziel aller anderen Fraktionen im Deutschen Bundestag, dass sie als eine „gleichgeschaltete Gesellschaft“ vom Nordkap bis Sizilien identifiziert, erteilt die AfD eine klare Absage. Europa bedeutet für uns die kraftvolle, belebende und kreative Auseinandersetzung seiner Nationen um die besten Wege und Resultate in Bildung und Wissenschaft, in der Wirtschaft und der Kultur. Dabei sieht sie die konkurrierenden europäischen Nationalstaaten in der Verpflichtung, die sich ergebenden Dispute im Sinne gegenseitiger Inspiration mit ausschließlich friedlichen Mitteln zu führen. Die Katastrophen des 20. Jahrhunderts dürfen sich nicht wiederholen. Sie haben Gesamteuropa in der Weltpolitik in vielen Bereichen marginalisiert. Wir erinnern uns dabei der Einschätzung des türkischen Staatsgründers Kemal Atatürk^1^: „Es mag eine Vielzahl von guten Kulturen in der Welt geben, aber es gibt nur eine Zivilisation – und das ist die europäische.“

Realpolitik im deutschen Interesse heißt, sich bei den großen globalen Herausforderungen stets auch des Nutzens für Deutschland zu vergewissern. Die AfD widerspricht daher der von der Bundesregierung unter Bundeskanzlerin Angela Merkel favorisierten Strategie einer regelbasierten, multilateralen Weltordnung. Dementsprechend kann es auch keine globale Bewältigung vielfältiger Probleme geben, sondern nur nationale Einzelfallentscheidungen, die bei Interessensgemeinsamkeiten auch mit anderen Staaten zusammen angegangen werden können. So lehnt die AfD das Pariser Klimaabkommen zwar ab, da sie den Klimawandel nicht als einen ausschließlich durch den Menschen verursachten ansieht. Sie folgt aber der Einschätzung der Wissenschaft, die eine weltweite Klimaveränderung feststellt, und tritt für internationale Strategien zur Abwehr der Folgen einer solchen ein.

Bei Problemen, die mehrere Nationen berühren, sollte Deutschland immer dann auf europäischer oder transkontinentaler Ebene Lösungen suchen, wenn es seinen Interessen dient und wenn diese am Ende des Abstimmungsprozesses untereinander auch gewahrt bleiben. So hätte es schon 2015 im vom Bürgerkrieg geplagten Syrien, wie von uns gefordert, die Einrichtung von Schutzzonen für die Zivilbevölkerung geben müssen, die durch ein robustes Mandat der Vereinten Nationen geschützt die massiven Flüchtlingsströme nach Europa, insbesondere nach Deutschland, verhindert hätten. Auch eine internationale Abstimmung im Kampf gegen den Terror liegt im deutschen Interesse. Die Bekämpfung des Coronavirus allerdings, bei der bislang sämtliche von der Europäischen Union koordinierten Maßnahmen versagt haben, ist vornehmlich eine nationale Herausforderung, die teilweise sogar nur regional bewältigt werden kann. Folglich hat jedes EU-Land noch heute seine eigene Anti-Corona-Strategie.

Ähnliches gilt auch für die sogenannte vierte industrielle Revolution. Die rasante Entwicklung Israels auf diesem Gebiet belegt, dass es eben kein supranationales Gebilde wie ein imaginiertes Vereinigtes Europa geben muss, um technologisch weltweit führend zu sein. Vielmehr fördert der Wettstreit der Nationen auch das Entwicklungstempo im Technologiebereich. Das ist allein daran erkennbar, dass in Deutschland 90 % aller Patente an mittelständische Unternehmen vergeben wurden, während große internationale Konzerne mit Stammsitz in Deutschland, so zum Beispiel Bosch, Siemens, BASF und andere, überwiegend als Patentverwerter auftreten; technologisch kann Deutschland durchaus noch, wenn auch nicht auf allen Gebieten, mit der wirtschaftlichen und militärischen Supermacht USA konkurrieren.

## Deutsche Souveränität: Transatlantisches Bündnis auf Augenhöhe

Die Besinnung auf die eigene Kraft in wirtschaftlicher und wissenschaftlicher Hinsicht gilt auch für ein stärkeres außenpolitisches Selbstbewusstsein. Realpolitik im deutschen Interesse heißt nichts anderes, als in Einzelfragen mit denen zusammen zu gehen, die unsere Anliegen unterstützen können. Das wäre zum Beispiel auch mit der ehemaligen US-Regierung unter Präsident Donald Trump möglich gewesen, da ein gemeinsamer Druck der USA und Deutschlands auf China in Sachen Patentschutz und Schutz des geistigen Eigentums größere Erfolgsaussichten gehabt hätte. Es gelang der Bundesregierung in unseren Augen bisher nicht, konzeptionell und tagespolitisch auf die schon zu Zeiten des ehemaligen US-Präsidenten Barack Obama eingeleitete Politikwende der USA hin zum pazifischen Raum zu reagieren. Gerade den vom ehemaligen US-Präsidenten Präsident Trump initiierten Rückzug aus den internationalen Verbots- und Reduzierungsverträgen über die Nuklearwaffenpotenziale hätte Deutschland zum Anlass nehmen müssen, auf eine stärker auf eigene und europäische Interessen ausgerichtete Verständigungspolitik mit Russland hinzuwirken. Hier hätte sich ein Vertrag der europäischen Nationen mit Russland über den Bann von nuklearen Trägersystemen mit einer Reichweite zwischen 550 und 5500 Kilometern („New INF Vertrag“) angeboten. Generell wäre Deutschland in der Lage, bei den europäischen Nachbarn auf deren stärkere Rolle im gemeinsamen NATO-Verteidigungsbündnis hinzuwirken. Dies setzt allerdings voraus, dass Deutschland erst einmal seinen finanziellen Selbstverpflichtungen gegenüber der NATO so nachkommt, dass ein deutlicher militärischer Mehrwert erkennbar ist. Die AfD unterstützt jegliches Bestreben, das die klassische Verteidigungsrolle der NATO reaktiviert. Die Alternative für Deutschland spricht sich aufgrund der katastrophalen Erfahrungen in Afghanistan, Mali und anderen Staaten gegen den Einsatz deutscher Truppen außerhalb des europäischen Verteidigungsraumes aus.

## Für Frieden und Stabilität in Europa: Neustart der deutsch-russischen Beziehungen

Deutschland ging es in Zeiten friedlicher Beziehungen zu Russland immer gut. Für Deutschland ist daher der Ausgleich mit Russland und die Integration des großen östlichen Nachbarn in das europäische Haus von größter Wichtigkeit. Blickt man auf die Phasen gelungener Kooperationen zwischen der Sowjetunion und Deutschland im letzten Jahrhundert zurück, so gingen dem politischen Interessenausgleich stets erfolgreiche Wirtschaftsbeziehungen voraus. Pragmatisch betrachtet muss man erkennen, dass Russland über die in Deutschland dringend benötigten Rohstoffe in mehr als ausreichenden Mengen verfügt, während man von Sankt Petersburg bis Wladiwostok großes Interesse an deutscher Technologie und Knowhow hat. Diese Situation gilt es zu beiderseitigem Vorteil zu nutzen. Dabei sind Vorhaltungen über unterschiedliche System- und Wertevorstellungen unangebracht. Einer klugen deutschen Außenpolitik gegenüber Russland war dies auch stets bewusst. Bereits 1975 wurde im Dekalog der Schlussakte der Konferenz über Sicherheit und Zusammenarbeit in Europa (KSZE) unter anderem das Prinzip der Nichteinmischung in die inneren Angelegenheiten anderer Staaten niedergelegt. Diesem Grundsatz sollte sich die deutsche Außenpolitik auch heute verpflichtet fühlen. Wir haben zu konstatieren, dass Russland nach jahrhundertelanger Zarenherrschaft und einer knapp 80 Jahre währenden Sowjetdiktatur nunmehr seit einer Generation auf dem Wege zur Demokratie ist. Auch in Deutschland gelang die Etablierung demokratischer Strukturen nicht ohne existentielle Gefährdungen, lange Unterbrechungen und heftige Rückschläge. Wir sollten mit einer unserer Demokratiegeschichte angemessenen Bescheidenheit Russland gegenübertreten und nicht beständig auf das zeigen, was in diesem großen Land noch nicht gelungen ist, sondern Russland die Zeit geben, die es auf seinem Weg benötigt. Wir sind fest davon überzeugt, dass es ohne eine gemeinsame Politik mit Russland weder Frieden noch Stabilität in Europa geben wird

## Im Wettbewerb mit China: Friedliche Kooperation oder Verdrängungskampf?

Ungleich schwieriger als im Fall Russland ist die außenpolitische Bewertung unserer Position gegenüber China. Das Reich der Mitte bedeutet für Deutschland Ansporn und Herausforderung zugleich, nicht ohne gelegentlich bedrohliche Akzente. Chinas massive Dominanz im afrikanischen Raum, seine Politik einer neuen Seidenstraße, deren Auswirkungen die europäische Wirtschaft spürt, und vor allem sein Streben nach Führung in Ostasien schaffen neue politische Kraftfelder. Ohne Zweifel wird das Seidenstraßenprojekt, das heute in Duisburg endet, einen Teil des weltweiten Warenverkehrs abdecken können. Offensichtlich ist aber auch, dass der Seetransport von Gütern preislich nicht zu unterbieten sein wird. Man stelle sich nur die über 20.000 Container eines dieser Tage zwischen China und Europa verkehrenden Containerschiffes verteilt auf die Waggons eines Güterzuges vor. Sichere maritime Handelsrouten sind für den internationalen Warenverkehr unerlässlich. Deutschland hat deswegen ein vitales Interesse an der Aufrechterhaltung und dem Schutz der internationalen Seewege, das es mit den USA, aber auch mit China teilt. Wir gehen davon aus, dass China sehr genau weiß, welche negativen Auswirkungen der Versuch nach sich ziehen würde, diese Handelsrouten einseitig kontrollieren zu wollen.

Kritisch sieht die AfD-Fraktion im Deutschen Bundestag das finanzielle und wirtschaftliche Engagement Chinas in Deutschland. Wie engagiert chinesische Unternehmen auf dem deutschen Markt agieren, zeigte 2016 die Übernahme der Kuka AG durch die chinesische Midea Group, die sich so den Zugang zum Wissen und zur Fertigungstechnologie eines Hochleistungsunternehmens auf dem Gebiet der Robotik sicherte. Für Deutschlands Zukunft als Wissenschafts‑, Technologie- und Fertigungsstandort ist es unerlässlich, den Ausverkauf deutscher Hochtechnologie zu verhindern und den chinesischen Begehrlichkeiten definierte Grenzen zu setzen. Gleichzeitig ist der chinesische Markt für deutsche Unternehmen so lukrativ geworden, dass wir beide Notwendigkeiten – hier das Ziel des langjährigen Exportweltmeisters Deutschland, seine Produkte in China anzubieten, dort die Sorge um den Verlust der technologischen Kernkompetenzen durch Firmenübernahmen – zu einem Interessenausgleich mit China bündeln müssen.

Deutschland sollte sich daher auf keinen Fall in die sich immer deutlicher abzeichnende Konfrontation der USA mit China hineinziehen lassen. Abstand nehmen sollte die Bundesregierung von Überlegungen, die deutsche Marine im asiatischen Raum operieren zu lassen. Abgesehen von der Problematik, mit welchen Schiffen die über Jahre ausrüstungstechnisch vernachlässigten deutschen Seestreitkräfte das bewerkstelligen sollen, stellt sich die ernste Frage, ob Deutschland aus Bündnistreue gegenüber den USA nicht plötzlich zu einem militärischen Akteur wider Willen in einer Region wird, in die es aus gutem Grund seit der Kaiserzeit keine Soldaten mehr entsandte.

## Deutschlands Renaissance als „ehrlicher Makler“

Deutschlands Bedeutung ist bei internationalen Gesprächen und bei den Konsultationen der Großmächte in den vergangenen Jahren deutlich geringer geworden. Die Anerkennung und Aufforderung des ehemaligen US-Präsidenten George Bush Senior gegenüber dem damaligen Bundeskanzler Helmut Kohl („Helmut, we are partners in leadership“) würde heute kein US-Präsident mehr wiederholen. Schlimmer noch: Wie einen Schüler, der disziplinlos und leichtfertig seine Talente vergeudet, kanzelte ein chinesischer Diplomat Deutschland nach dem Ende seiner temporären Mitgliedschaft im UN-Sicherheitsrat im Dezember 2020 ab: „Das Auftreten Deutschlands im Sicherheitsrat hat nicht den Erwartungen der Welt und den Erwartungen des Rates entsprochen.“ Der russische Vertreter ergänzte unmissverständlich: „Sie werden uns nicht fehlen.“^2^ Damit rückt auch der lange gehegte Wunsch Berlins, dauerhaft Mitglied im UN-Sicherheitsrat zu werden, in weite Ferne. Mögen wir als immer noch starke Wirtschaftsmacht im Kreis der G20-Staaten eine durchaus wahrnehmbare Stimme haben, droht uns im internationalen Völkerparlament der UNO der Verweis auf die hinteren Plätze. Durch Deutschlands rasche, nicht selten unberechenbare Positionierung bei internationalen Konfliktlagen, wie in der Ukraine, wie jetzt in Weißrussland oder zuvor in Venezuela, droht der Verlust des guten Rufes auf dem internationalen Parkett.

Deutschland trägt nur sehr wenige koloniale Bürden und fällt als Mittelmacht militärisch nicht ins Gewicht; Deutschland könnte als Vermittler, als ehrlicher Makler international entscheidend zur Konfliktentschärfung und Befriedung beitragen. So wie es Bismarck 1878 gelang, die Interessen der europäischen Mächte auf dem Balkan so auszubalancieren, dass alle ihre Ansprüche berücksichtigt sahen, so könnte Deutschland seine Rolle unter den Nationen der Welt verstehen. Dafür wäre allerdings ein Paradigmenwechsel im Auswärtigen Amt notwendig, der mit der oben erwähnten Rückkehr zur klassischen Diplomatie einhergeht und auf eine Einflusspolitik in Menschenrechts‑, Klima‑, Gender- und Frauenfragen verzichtet.

Die deutsche Außenpolitik sollte sich mit diesem Selbstverständnis besonders dort einbringen, wo wir traditionell auf guten Beziehungen aufbauen können. Dies gilt für Weißrussland ebenso wie für die Ukraine und die anstehenden Neuverhandlungen mit dem Iran zur Wiener Nuklearvereinbarung über das iranische Atomprogramm (JCPoA). Einen wichtigen Raum nehmen die zunehmend schwierigeren Beziehungen zur Türkei ein. Während Deutschland lange Zeit ein weitgehend problemloses Verhältnis zur Türkei pflegte und noch unter Helmut Kohl im Gespräch mit den türkischen Partnern auf die türkische Politik einwirken konnte, nutzt heute Präsident Recep Erdogan seine Landsleute in der Bundesrepublik zur Einflussnahme auf die deutsche Politik.

Deutschlands schwindende internationale Bedeutung offenbart sich angesichts der militärischen Interventionspolitik der Türkei in Syrien, Libyen, Aserbaidschan oder gar Mali in besonderem Maße: Deutschland verfügt kaum noch über Möglichkeiten, Nennenswertes zur Vermittlung zwischen den Konfliktbeteiligten beizutragen.

In den Syrienkonflikt ist Deutschland direkt eingebunden. Knapp eine Million syrische Flüchtlinge leben in Deutschland, politische Entwicklungen dort wirken unmittelbar nach Deutschland hinein. Festzustellen ist, dass sich die auswärtige Politik der Bundesregierung aufgrund ihrer rigorosen Ablehnung eines direkten Kontakts zu Präsident Baschar al-Assad selbst der Chancen beraubt, in welcher Weise auch immer, die Situation in Syrien zum Besseren zu wenden und den syrischen Flüchtlingen hierzulande die Aussicht auf eine Rückkehr in ihr Heimatland aufzuzeigen. Sinnvoll wäre es dagegen, durch Verhandlungen eine verbriefte Zusage des syrischen Präsidenten zu erreichen, die heimkehrenden Flüchtlinge aus Deutschland weder zu verfolgen noch ihren Immobilienbesitz zu enteignen. Als Kontrollorgan könnte hier ein „Special Envoy“ der UN dienen. Die AfD-Fraktion im Deutschen Bundestag hat dafür den früheren stellvertretenden UN-Generalsekretär Klaus Töpfer vorgeschlagen.

Da nach dem vermeintlichen arabischen Frühling der gesamte arabische Raum zum Teil völlig destabilisiert ist, liegt es nach Ansicht der AfD-Bundestagsfraktion auch im deutschen Interesse, das in Gang zu setzten, was in den 1970er-Jahren durch den KSZE-Prozess zur Aufweichung und völligen Verschiebung damals starrer Situationen geführt hat. Die Abgeordneten der AfD-Fraktion im Deutschen Bundestag sind wie einige Stimmen aus der Union und aus den Reihen der Grünen der Überzeugung, dass die internationale Staatengemeinschaft eine Konferenz über Sicherheit und Zusammenarbeit im Nahen und Mittleren Osten unter Einbeziehung Israels initiieren sollte. Uns ist dabei bewusst, dass dies mit einigen großen Treffen aller Beteiligten allein nicht getan ist. Die Schlussakte von Helsinki stand am Ende eines viele Jahre dauernden Prozess. Wir sind aber der Überzeugung, dass es dieses Kraftaktes bedarf, um in jener Region eine weitere Verschärfung der Konflikte und das Entstehen neuer Auseinandersetzungen zu verhindern.

## Fazit: Neue Instrumente für eine erfolgreiche deutsche Außenpolitik

Um als deutsche Bundesregierung die zuvor erwähnten vielfältigen Herausforderungen der deutschen Außenpolitik meistern zu können, ist unseres Erachtens eine umfassende Reform der deutschen auswärtigen Politik vonnöten. Dass die parlamentarische Kontrolle der Außen- und Sicherheitspolitik der Bundesregierung inzwischen nur noch eine marginale Rolle spielt, ist beklagenswert genug. Dass wir aber durch immer neue Restriktionen unseren Auslandsgeheimdienst (BND) fast wirkungslos machen, wiegt da schon sehr viel schwerer. Die AfD-Fraktion im Deutschen Bundestag fordert deshalb schon seit längerem die Einrichtung eines nationalen Sicherheitsrates als ständig tagendes Gremium. Ähnlich wie der National Security Council in den USA sollte ein deutscher nationaler Sicherheitsrat die Expertise aus den entsprechenden Ministerien mit der taktischen Umsetzung durch die Exekutiv- und Hilfsorgane im Inneren und Äußeren abstimmen und koordinieren. Durch eine permanente und feststehende Organisationsstruktur ist der rasche Abgleich nationaler und internationaler Informationen möglich und gewährleistet so die schnelle und zeitnahe Entwicklung nationaler Handlungsoptionen.

## Footnotes

1 Zit. n. Rill, B. (1985). *Kemal Atatürk* (S. 80). Hamburg: Rowohlt.

2 Zit. n. Der Spiegel (2020, 17. Dez.). Russland an Deutschland: »Sie werden uns nicht fehlen«. https://www.spiegel.de/ausland/china-und-russland-attackieren-deutschland-im-uno-sicherheitsrat-a-6d271d73-457e-43d2-9b13-d53eeec93995. Zugegriffen: 27. Mai 2021.

